# Lower-limb locomotor function studies using walking speed as an assessment indicator: A bibliometric review from 2014 to 2024

**DOI:** 10.1097/MD.0000000000042756

**Published:** 2025-06-13

**Authors:** Luming Yang, Yuan Liu, Mei Wu, Xinye Liu, Longbin Zhang, Shiyang Yan

**Affiliations:** a National Engineering Research Center of Clean Technology in Leather Industry, Sichuan University, Chengdu, China; b Laboratory of Intelligent Clothing & Sport Biomechanics, Sichuan University, Chengdu, China; c West China Fourth Hospital, Sichuan University, Chengdu, China; d KTH MoveAbility Lab, Department of Engineering Mechanics, KTH Royal Institute of Technology, Stockholm, Sweden.

**Keywords:** aging, knee osteoarthritis, lower-limb locomotor function, rehabilitation training, walking speed

## Abstract

**Background:**

Walking speed is an important kinematic parameter for evaluating lower-limb locomotor function. Our review aims to identify the research trends and hotspots of walking speed in assessing mobility across various populations through bibliometric methods.

**Methods:**

A total of 1656 articles on the topic of walking speed published between 2014 and 2024 were retrieved from the Web of Science core database. CiteSpace was used for bibliometric visualizations, analyzing key indicators, such as the number of annual publications and their citation frequency, institutions, research areas, co-cited references, keywords, and other indicators.

**Results:**

The number of studies on walking speed has consistently increased in the past decade. The University of Delaware publishes the most articles (37 articles). Research areas indicate that walking speed has been widely used in orthopedics, neuroscience, and rehabilitation. Recent studies are closely associated with gait analysis, aging, and youth populations through co-cited reference analysis. Moreover, keyword results show that knee osteoarthritis, physical activity, and muscle strength are the primary research topics. Body function and sex differences have become the latest research hotspots.

**Conclusion:**

Walking speed is widely used to evaluate the activity ability of people with movement disorders and their exercise rehabilitation effect, especially in older adults. We hope to provide an overview of walking speed research for researchers in geriatrics, rehabilitation medicine, and biomedical engineering, encouraging them to focus on analyzing the relationship between walking speed and the mechanism of musculoskeletal system movement in future studies.

## 1. Introduction

Walking is the primary mode of human locomotion. Walking speed can reflect various performance characteristics, including motor control, musculoskeletal status, cardiopulmonary endurance, and cognitive ability, referring to the “sixth vital sign.”^[1–3]^ It is significant in gait studies on sports biomechanics, rehabilitation medicine, and geriatrics, serving as a valuable tool for assessing an individual’s health status, risk of disease, and survival rates.^[4–6]^

Clinically significant walking speeds include self-selected walking speed (SSW) and maximum walking speed (MWS).^[3]^ SSW is stable and repeatable, reflecting participants’ typical daily gait.^[2,7]^ The MWS task requires greater requirements in motion control and is more sensitive than SSW for assessing lower-limb strength and coordination.^[3,8]^ For example, it performs better classification than SSW in evaluating cerebrospinal fluid tap test efficacy in patients with idiopathic normal pressure hydrocephalus.^[9]^ Both speeds can effectively reflect the exercise ability of the subjects, particularly the lower-limb function.

The study of walking speed encompasses a wide range of populations, with significant differences in walking speed characteristics among different sexes, ages, degrees of illness, and occupations.^[6,10–13]^ From an age perspective, children typically begin to walk independently between 8 and 16 months.^[14,15]^ Their walking speeds gradually increase, stabilizing from age 9 to 17 to a nearly mature pace.^[16]^ After age 11, there is a sex difference, with boys walking about 0.24 m/s faster than girls.^[17]^ Researchers in the United States, the United Kingdom, and China have developed regional clinical walking speed norms and reference values for children.^[16–18]^ Adults achieve a stable speed of 1.3 to 1.5 m/s around 20 years old, significantly decreasing after 60.^[12,13,18]^ Furthermore, walking speed is frequently used to classify the degree of motor dysfunction in patients. For instance, researchers stratified a sample of children with cerebral palsy by walking speed to ensure that participants were adequately represented in the Gross Motor Function Classification System.^[19,20]^ The Asian Working Group for Sarcopenia recommends a walking speed of 1.0 m/s as the cutoff for the low physical performance of completing a 6-meter walk test.^[21]^ Additionally, the European Working Group on Sarcopenia in Older People recommended that the walking speed of completing the 4-meter test ≤ 0.8 m/s as an indicator of severe sarcopenia.^[22]^

Walking speed is closely linked to coordination and efficiency of lower-limb locomotion, a critical research parameter in sport sciences. Currently, there is no comprehensive review of walking speed research. Therefore, our study aims to utilize bibliometric methods to analyze the current research status of walking speed as a clinical index for rehabilitation in evaluating lower-limb motor ability by integrating studies of different groups. Furthermore, we provide key collaboration structures (e.g., countries, institutions, and authors), research objects, and research hotspots in walking speed research over the past decade for researchers through relationship networks and knowledge maps, offering a scientific and valuable reference for evaluating the clinical exercise ability and rehabilitation effect.

## 2. Methods

### 2.1. Data source

**Figure 1.**
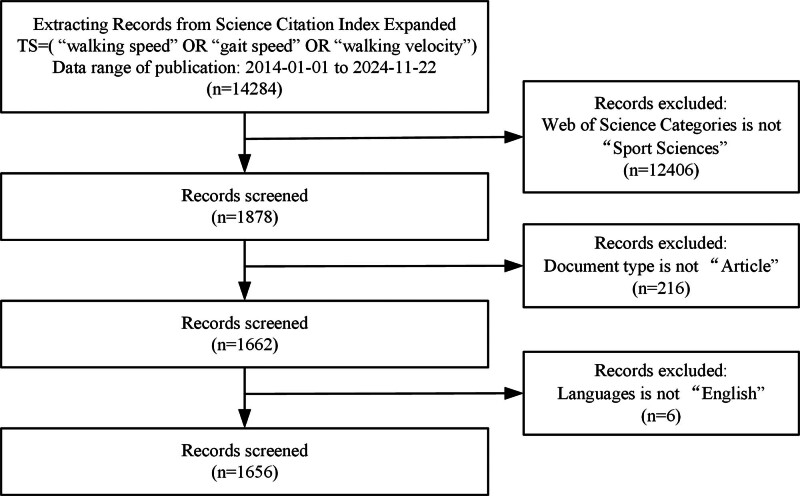
Flowchart of document selection.

We searched the Science Citation Index Expanded within the Web of Science Core Collection using the search string TS = (“walking speed” OR “gait speed” OR “walking velocity”). The search covered publication dates from January 1, 2014, to November 22, 2024. Articles were included if they met the following criteria: (1) The research field was limited to “Sport Sciences.” (2) The document type was specified as “Article.” (3) Articles were published in English. The article screening process is illustrated in Fig. 1, resulting in a final dataset of 1656 articles. This bibliometric study did not involve human participants or animals and thus did not require ethical approval.

### 2.2. Analytical tools and methods

Bibliometric data were analyzed using CiteSpace 6.1.1. All 1656 articles were imported into the software, with the document type restricted to “Article” to exclude records with incomplete or duplicate information. The dataset included 1656 articles and 35,921 co-cited references, with the time-slicing interval set to 1 year. The log-likelihood ratio test was used to extract clusters based on keywords from the articles. Origin 2023 was used to visualize temporal trends in publication counts.

## 3. Results

Clustering results are evaluated using modularity *Q* measures (*Q* values) and a silhouette metric (*S* values). *Q* > 0.3 indicates the clustering network structure is obvious, and *S* ≥ 0.7 indicates the clustering results are reliable and intuitive.^[23]^ This study’s *Q* and *S* values were 0.4 and 0.7, respectively.

### 3.1. Trends in annual publications and citations

**Figure 2.**
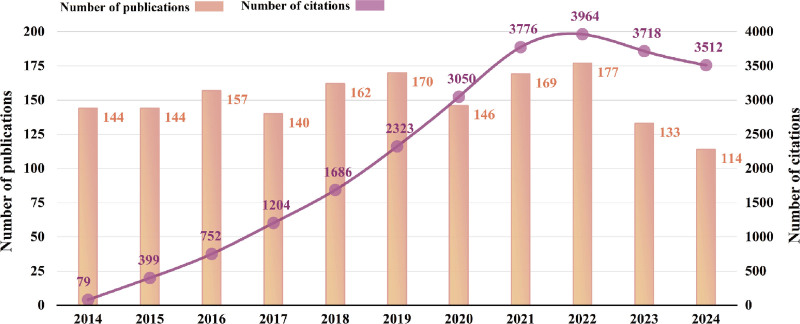
Annual trends in publications and citations.

The statistics for the number of publications and citations are shown in Fig. 2. The number of publications in this field shows a fluctuating upward trend between 2014 and 2022, followed by a slight decline after 2023. Citation counts show a marked overall increase, peaking in 2022.

### 3.2. Analysis of countries and institutions

**Figure 3.**
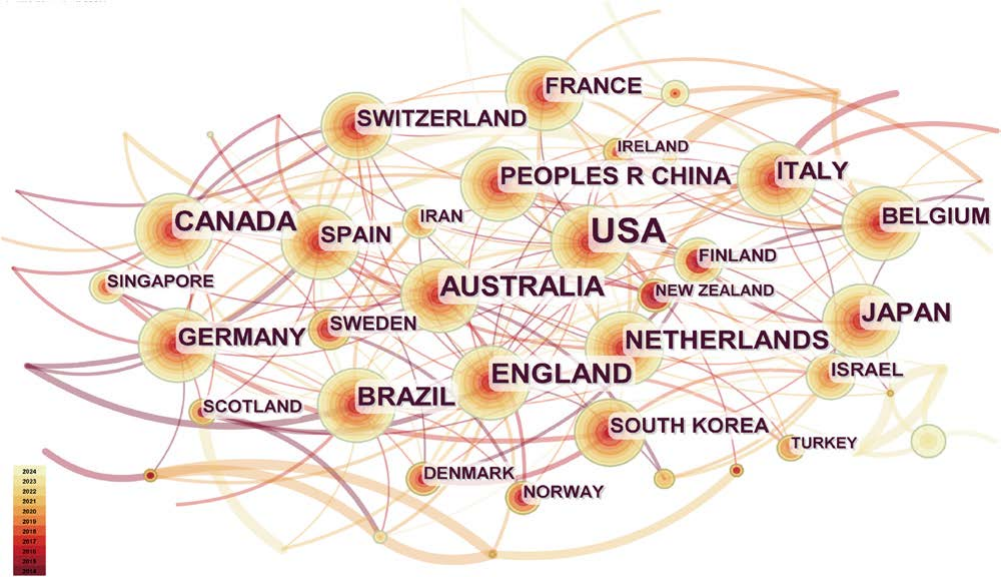
Visualization network map of the cooperation relations among countries.

A cooperative network map of international research on human walking speed is shown in Fig. 3. The size of each node in the graph corresponds to the frequency of the represented content.^[24]^ The connection thickness between country nodes indicates the frequency of cooperation.^[25]^ Many countries cooperated, and the published articles primarily focus on developed countries. Table S1, Supplemental Digital Content, https://links.lww.com/MD/P145, lists the countries publishing more than 100 research articles. The United States accounts for 39.4% of the publications, demonstrating significant academic impact. Brazil (7^th^ place, 91 articles) and China (8^th^ place, 84 articles) have shown increasing research activity in recent years.

**Figure 4.**
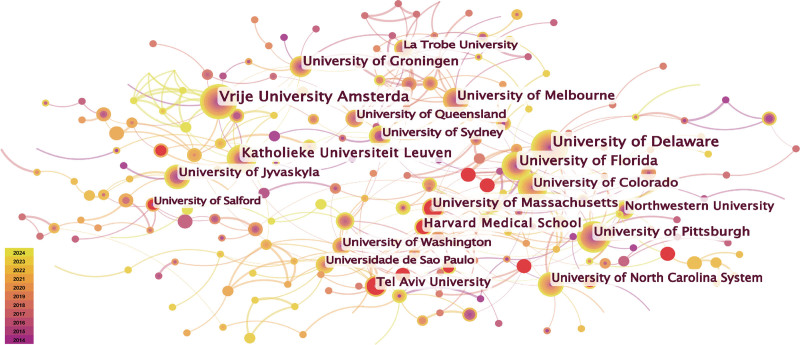
Visualization network map of the cooperation relations among institutions.

A network map of the collaborative research institutions is shown in Fig. 4, demonstrating the close collaboration between major institutions. Red nodes indicate that institutions have experienced a significant increase in published articles. In recent years, Harvard Medical School, the University of Massachusetts, and Tel Aviv University have emerged as emerging research institutions. Table S2, Supplemental Digital Content, https://links.lww.com/MD/P146, lists the institutions publishing more than 20 articles. The University of Delaware publishes the most papers, focusing on the motor function and rehabilitation effects of patients with stroke, concussion, knee osteoarthritis (KOA), chronic low back pain, and concussion.

### 3.3. Analysis of fundings

**Figure 5.**
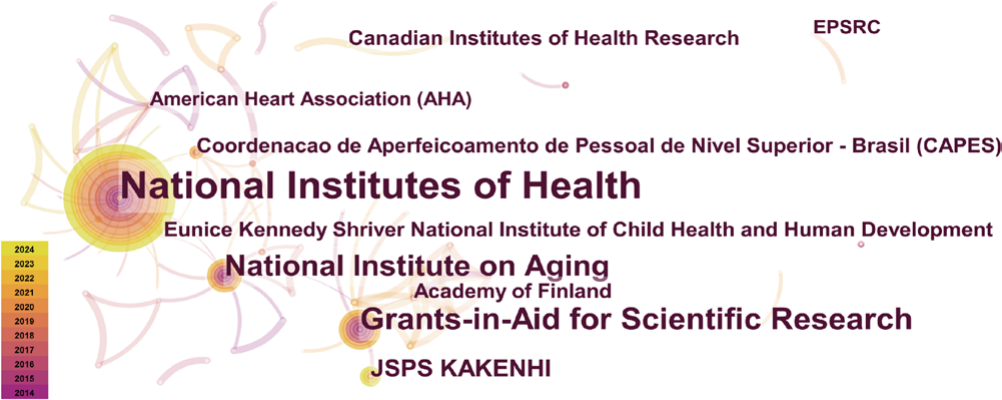
Visualization network map of funds.

A network map of funding is shown in Fig. 5. Most research fundings originate in developed countries. The top 5 funding sources with the highest frequency of contributions are the United States (3) and Japan (2). Among them, the National Institutes of Health (83 times, 14.6%) makes significant scientific contributions and is one of the world’s largest biomedical research funding agencies.

### 3.4. Analysis of research areas

**Figure 6.**
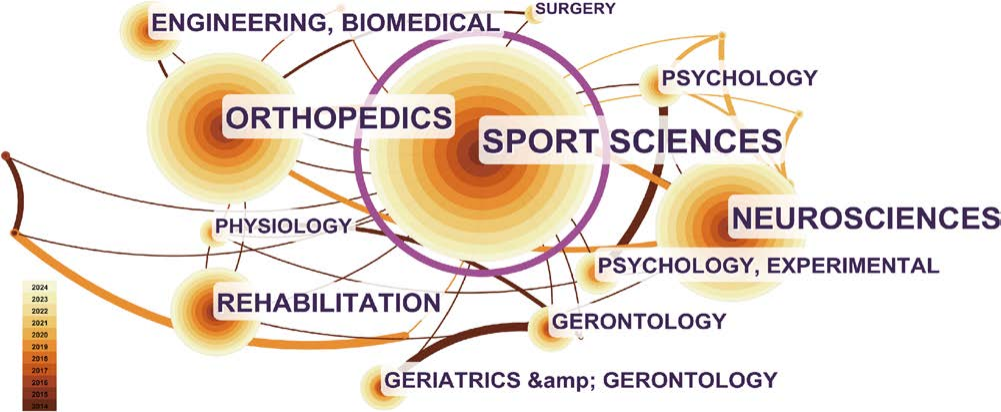
Visualization network map of research areas.

Research area analysis helps researchers understand the domain diffusion breadth of walking speed research.^[26]^ A visualization network of research areas is shown in Fig. 6. Excluding search topics, the top 5 research areas associated with walking speed are “Orthopedics” (51.2%), “Neuroscience” (47.5%), “Rehabilitation” (20.1%), “Biomedical Engineering Biomedical” (10.7%), “Psychology” (6.49%).

### 3.5. Analysis of journals

A visualization network of the co-cited journals is shown in Fig. S1, Supplemental Digital Content, https://links.lww.com/MD/P148. Tables 1 and 2 list the top 5 journals in terms of publications and co-citations. The journal *Gait & Posture* has the highest number of publications (39.3%) and citations, publishing a large number of walking speed studies on gait analysis, posture control, and rehabilitation medicine.

**Table 1:** The information of the top 5 journals.

Ranking	Frequency	Journal	IF (2023)	Cited frequency	Non-self citation frequency
1	651	Gait Posture	2.2	17,404	16,230
2	148	Archives of Physical Medicine and Rehabilitation	3.6	25,817	25,131
3	136	Clinical Biomechanics	1.4	9077	8815
4	95	Journal of Aging and Physical Activity	1.4	2569	2441
5	83	Human Movement Science	1.6	5486	5355

**Table 2:** The information of the top 5 co-citation journals.

Ranking	Frequency	Co-citation journal	IF (2023)	Cited frequency	Non-self citation frequency
1	1227	Gait Posture	2.2	17,404	16,230
2	911	Archives of Physical Medicine and Rehabilitation	3.6	25,817	25,131
3	762	Journal of Biomechanics	2.4	30,780	20,037
4	663	Physical Therapy	2.5	14,926	14,567
5	627	Clinical Biomechanics	1.4	9077	8815

### 3.6. Analysis of authors

**Figure 7.**
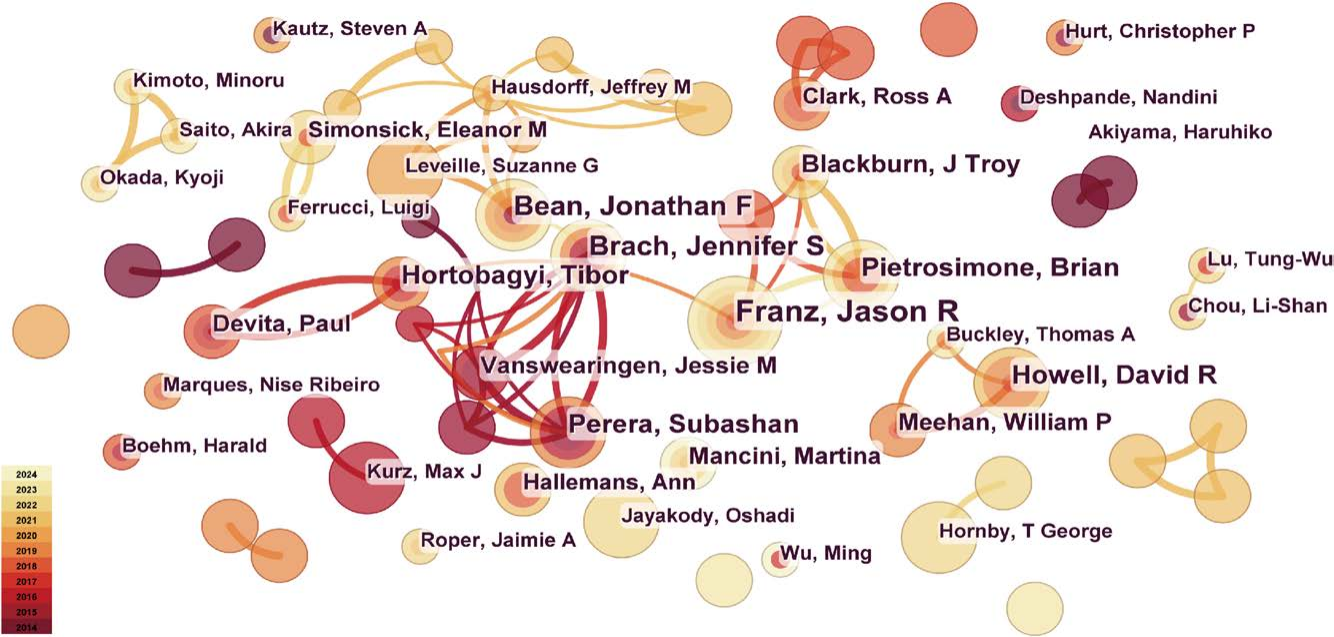
Visualization network map of the cooperation relations among authors.

The collaboration network among authors is shown in Fig. 7. Cooperation between researchers is relatively scattered and primarily based on small-scale collaboration. Table S3, Supplemental Digital Content, https://links.lww.com/MD/P147, lists the authors with 8 or more published articles. Their researches predominantly focus on geriatric rehabilitation and neurological impairment. Notably, Franz Jason R. from the University of North Carolina published 11 articles (115 citations) that contributed significantly to biomechanics and mobility studies in older adults.

### 3.7. Analysis of co-cited references

**Figure 8.**
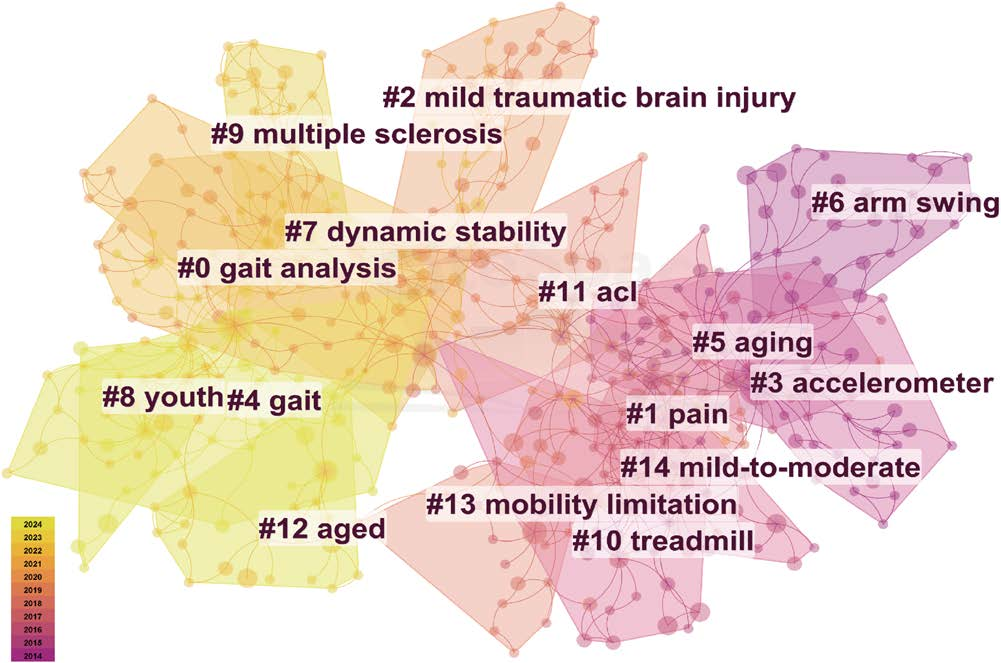
Cluster analysis of co-cited references.

Co-cited references analysis examines the correlations between citations.^[27]^ It can help researchers identify research trends, research foundations and key principles within a particular academic field.^[23,28]^ The cluster mapping of co-citation literature is shown in Fig. 8. The smaller the cluster number, the more keywords it includes. Therefore, clusters with smaller numbers are more representative.^[25]^ The top 5 largest clusters are labeled “gait analysis,” “pain,” “mild traumatic brain injury,” “accelerometer,” and “gait.” The most recently generated clusters are “aged,” “youth,” and “gait,” which were published on average from 2019 to 2020.

Table 3 lists the top 5 frequently co-cited references in walking speed studies. A higher citation frequency highlights the significance of the literature in walking speed foundational research. (1) Fukuchi et al conducted a systematic review investigating the influence of walking speed on gait biomechanics in healthy individuals aged 4 to 85 years.^[29]^ Their analysis demonstrated a significant moderating effect of walking speed on gait patterns and emphasized the critical role of walking speed differences when comparing pathological and healthy gait. (2) Studenski et al conducted a follow-up study on the relationship between gait speed and survival time in older adults, indicating that predicting survival based on walking speed is plausible.^[4]^ (3) Cruz-Jentoft et al updated the definition and diagnostic criteria for sarcopenia, identifying walking speed ≤ 0.8 m/s as a criterion for diagnosing severe sarcopenia.^[22]^ (4) Middleton et al established standards for gait test design, walking speed cutoffs, and corresponding predictive results.^[1]^ (5) Morris et al showed that wearable sensors effectively measure walking speed in healthy individuals and Parkinson patients, laying a foundation for future research in this area.^[30]^

**Table 3:** The information of the top 5 co-cited references with the highest citation frequency.

Ranking	Frequency	Year	Author	Title	Citations	Literature sources
1	33	2019	Fukuchi CA. et al.	Effects of walking speed on gait biomechanics in healthy participants: A systematic review and meta-analysis	281	Systematic Reviews DOI 10.1186/s13643-019-1063-z
2	28	2011	Studenski S. et al.	Gait speed and survival in older adults	3314	Journal of the American Medical Association DOI 10.1001/jama.2010.1923
3	20	2019	Cruz-Jentoft AJ. et al.	Sarcopenia: Revised European consensus on definition and diagnosis	9733	Age and Ageing DOI 10.1093/aging/afy169
4	18	2015	Middleton A. et al.	Walking speed: The functional vital sign	858	Journal of Aging and Physical Activity DOI 10.1123/japa.2013-0236
5	12	2019	Morris R. et al.	Validity of mobility lab (version 2) for gait assessment in young adults, older adults and Parkinson disease	134	Physiological Measurement DOI 10.1088/1361-6579/ab4023

### 3.8. Analysis of keywords

**Figure 9.**
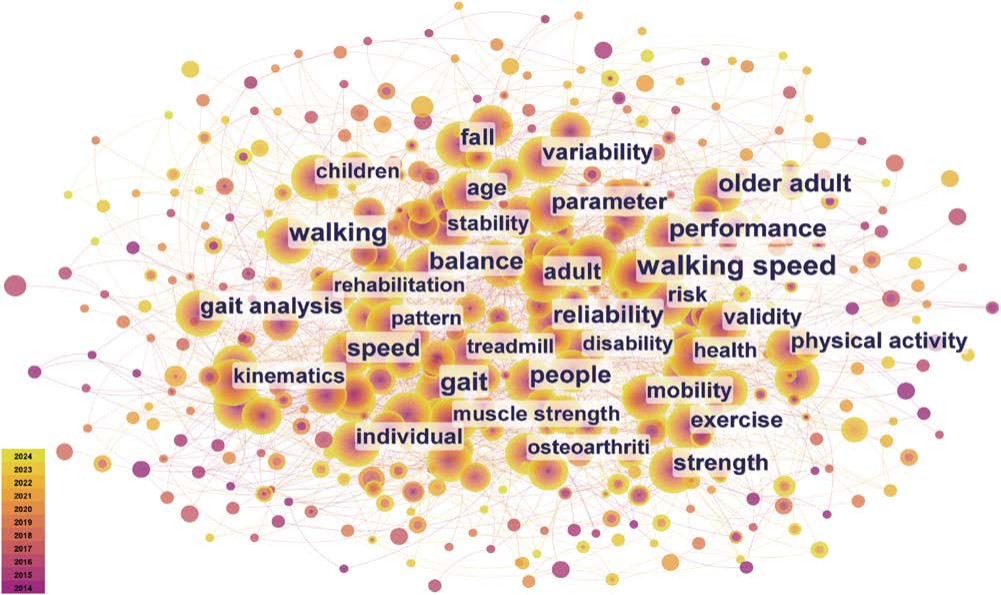
Visualization network map of keywords.

Keyword analysis can help researchers quickly identify influential keywords and understand content and trends in particular research fields. A network map of the keywords is shown in Fig. 9. This can be used to identify research hotspots. Excluding our search terms, the ten keywords with the highest frequency are “gait” (260 cases), “performance” (228 cases), “older adult” (208 cases), “balance” (197 cases), “reliability” (196 cases), “people” (188 cases), “fall” (146 cases), “parameter” (138 cases), “gait analysis” (131 cases), “variability” (131 cases).

**Figure 10.**
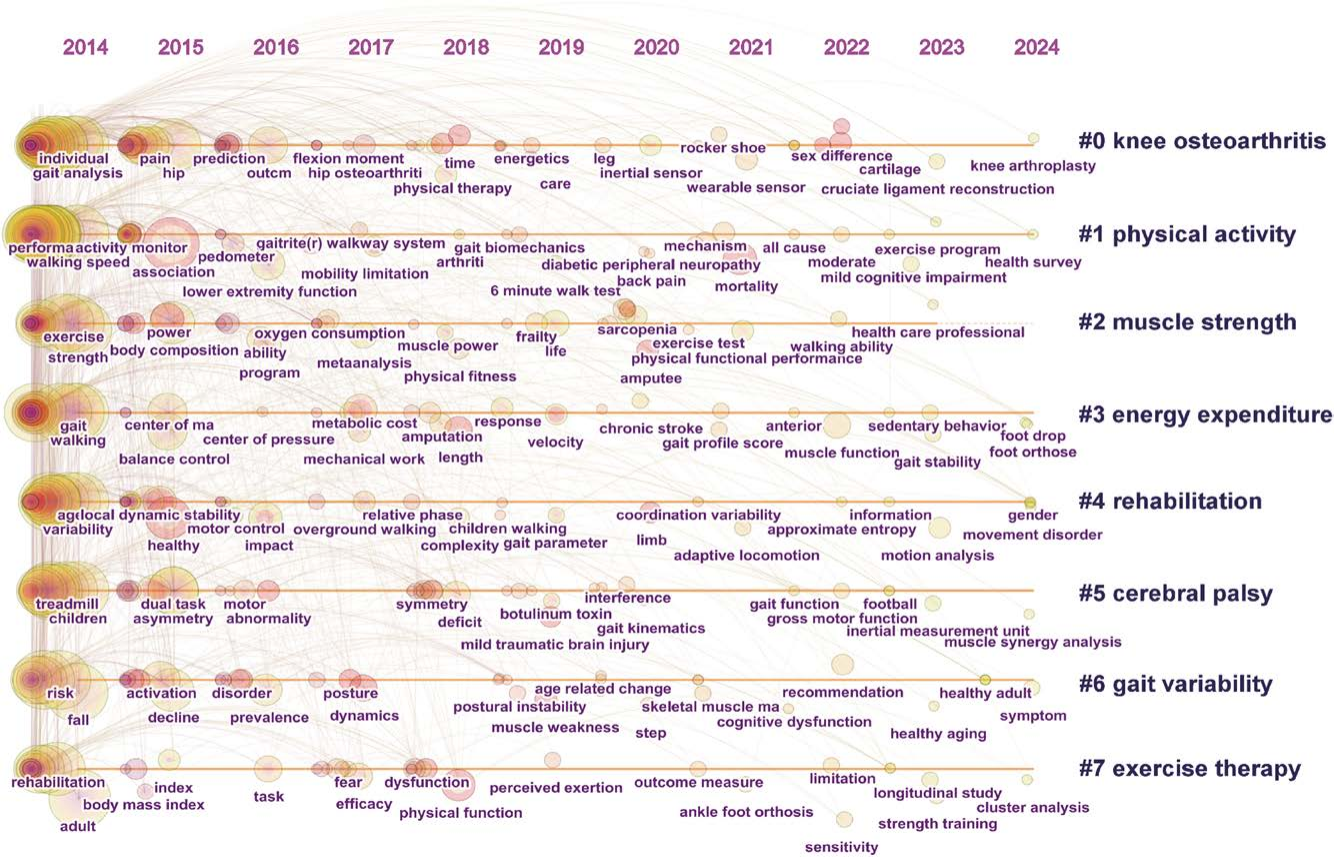
Cluster timeline diagram of the keywords.

The timeline of keyword clustering is shown in Fig. 10, emphasizing temporal relationships.^[24]^ This provides insights into trends in research hotspots related to “walking speed” as a keyword. In the 3 most representative clusters, the relationship between walking speed parameters and KOA, physical activity, and muscle strength was studied. (1) “Knee osteoarthritis” (Cluster 0): From 2014 to 2017, studies focused on gait analysis and hip osteoarthritis. Between 2018 and 2021, the focus shifted to wearable devices, sensors, and lower-limb care. After 2022, research concentrated on muscle moment arm and clinical treatments, such as knee arthroplasty and cruciate ligament reconstruction. (2) “Physical activity” (Cluster 1): From 2014 to 2017, studies focused on lower-limb locomotor function and disorders. Between 2018 and 2021, the focus shifted to validating the effectiveness of the testing process and exploring the relationship between motor function and disease. After 2022, research hotspots included sports, cognitive disorders, and health surveys. (3) “Muscle strength” (Cluster 2): From 2014 to 2017, studies focused on muscle strength, exercise patterns, and meta-analysis. Between 2018 and 2021, sarcopenia, amputation, and physical functional performance became key focus areas. After 2022, attention shifted to walking ability and professional clinical care.

**Figure 11.**
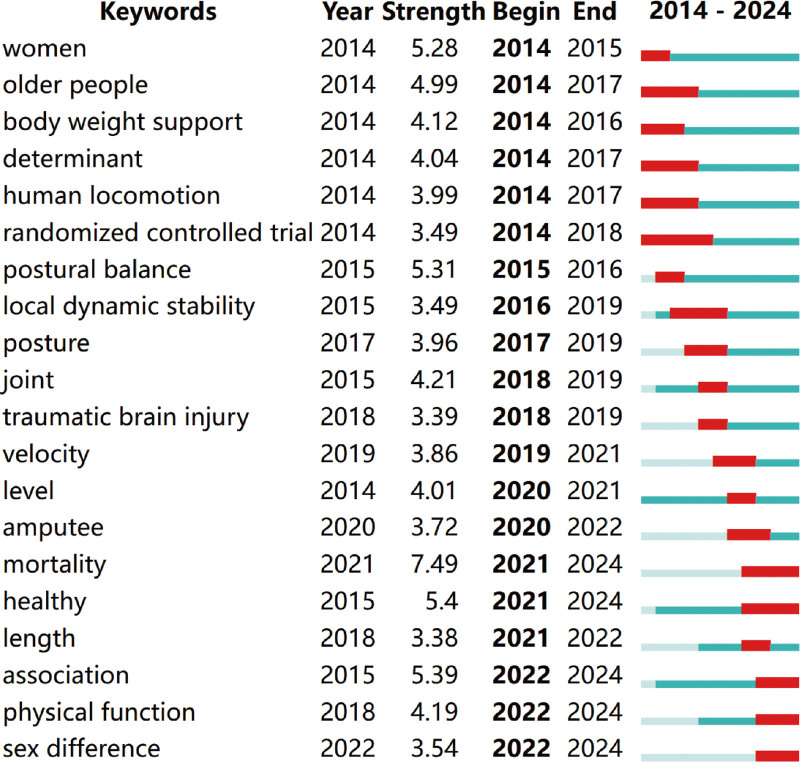
Top 20 keywords with the strongest citation bursts.

Keyword burst analysis helps researchers identify emerging research hotspots and changes in particular research fields. The 20 keywords with the strongest citation bursts are shown in Fig. 11. The top 5 keywords with the strongest bursts, in descending order, are “mortality,” “healthy,” “association,” “postural balance,” and “women,” with bursts strength ranging from 7.49 to 5.28. Emerging topics between 2022 and 2024 include “association,” “physical function,” and “sex difference.”

## 4. Discussion

We conducted a bibliometric analysis of studies on walking speed in the Sport Sciences from 2014 to 2024, including 61 countries, 2147 research institutions, 6955 researchers, and 35,921 citations. The frequency of citations for this research topic rose from 79 in 2014 to 3512 in 2024, marking an increase of over 43 times. This trend highlights the growing significance of walking speed research in sport sciences. Therefore, it is meaningful to review it. The study of walking speed is closely related to orthopedics, neuroscience, and rehabilitation. Co-cited references analysis elucidates the research foundation, primarily focusing on gait analysis, aging, neural injury, pain and children. Furthermore, based on the results of 3 essential kinds of keyword analyses, we extracted walking speed as a critical clinical indicator, which is primarily utilized to characterize lower-limb movement disorders and assess the efficacy of lower-limb rehabilitation training. Diseases and aging are the primary contributors to motor disabilities.^[31]^ The typical groups studied were older adults (38.6% of the articles) and patients with movement disorders. KOA is one of the most extensively studied diseases (10.0% of the articles).

### 4.1. Walking speed characterizes lower-limb motor disabilities

Walking speed serves as a critical indicator for assessing muscle activation, joint range of motion, and neural control in individuals with functional disorders. It can also be utilized to predict the severity of movement impairments in patients.

Walking speed decreases with age, often owing to a natural decline in muscle strength and neurological control.^[32–35]^ Physiologically, impaired leg propulsive in older adults is associated with weakened muscles and reduced forward propulsion forces. Franz et al showed that older adults lacking ankle strength required greater hip moments to initiate leg swings than younger adults.^[36]^ Weakness in the hip extensor and ankle flexor muscles reduces forward propulsion during walking, leading to declines in self-selected and MWSs.^[37–39]^ Similarly, the thickness of the vastus lateralis affects the force during the push-off and landing phases, which are positively correlated with walking speed.^[40]^ Reduced limb coordination caused by aging is one of the internal reasons for decreased walking speed.^[41]^ In terms of cognitive ability, Ojagbemi et al found that cognitive decline adversely affects walking speed by establishing a linear regression model and longitudinal analysis.^[42,43]^ Sensory impairments, such as hearing loss, were also associated with reduced walking speed in older adults.^[44]^

Knee osteoarthritis, a degenerative joint disease, is associated with significant reductions in walking speed as the condition progresses.^[45]^ Limited range of motion, joint pain, muscle weakness, and psychological barriers collectively contribute to locomotor dysfunction.^[46,47]^ Bungo et al identified quadriceps tendon stiffness as a factor restricting gait swing and reducing walking speed in patients with KOA,^[48]^ while quadriceps muscle weakness also negatively affects walking speed.^[49]^ Walking speed is a prognostic indicator of locomotor function and joint health.^[50,51]^ Studies have shown that patients with KOA have a 104% increased risk of knee replacement surgery for a 0.1 m/s decrease in walking speed.^[52]^ A slow walking speed of <1.2 m/s in the standard walking test is a threshold for assessing the risk of death in patients with radiographic KOA.^[53]^

### 4.2. Walking speed assesses the effectiveness of lower limb motor rehabilitation

Walking speed is one of the main indicators to evaluate the rehabilitation effect, which can be used to reflect the improvement of muscle strength, joint flexibility and posture stability in rehabilitation training program.

Exercise interventions to improve lower-limb locomotor function in older adults include strength training and sports training. Strength training represents a fundamental category of sports rehabilitation methodologies, encompassing a diverse array of specific training methods. Resistance training can effectively improve lower-limb muscle strength in older adults.^[54]^ Functional strength interventions, such as suspension exercises,^[55]^ flywheel resistance training and interval-walking training can significantly activate muscles in older adults and increase their walking speed.^[56,57]^ Low-load high-repetition resistance training or high-intensity interval training can also significantly increase the walking speed of older adults with movement disorders.^[58,59]^ Sports training is another typical exercise rehabilitation method used by older adults. It can improve walking speed by improving their cognition, posture stability, and strength. Traditional horseback riding can strengthen the knee extension muscles,^[60]^ whereas Pilates and Nordic walking can increase the walking speed.^[61,62]^ Moreover, dance could improve cognitive and motor dysfunction.^[63,64]^

Physical exercise positively affects KOA patients both pre- and post-surgery.^[65]^ In the early stages of the disease, walking exercises can enhance the strength of the knee extension muscles.^[66]^ High-intensity resistance aquatic training is used to improve the patient’s walking speed.^[67]^ Concurrently, a multicomponent exercise program was used to increase patients’ daily and MWSs.^[68]^ Psychological motivation can further enhance the effects of rehabilitation training on KOA patients. Training with a hybrid assistive limb robot can facilitate earlier recovery of walking ability.^[69]^ Additionally, twelve weeks of elastic resistance exercise after total knee replacement can improve muscle mass and positively affect walking speed.^[70]^

### 4.3. Frontiers and outlook

Walking speed is a critical indicator of motor ability in older adults and individuals with motor dysfunction, and its clinical value has been widely recognized. Previous studies have primarily used statistical analysis to quantitatively describe the walking speed characteristics of participants with lower-limb functional dysfunction, such as correlations between walking speed and leg muscle strength or the postsurgical recovery effect.^[71]^ With the advancements in experimental equipment and testing protocols, research has expanded to examine the synergistic effects of cognitive ability, neural control, and the musculoskeletal system on motor function. However, the mechanisms and functional relationships between these factors and walking speed require further elucidation to enhance their accuracy as signals of motor ability and to help doctors provide timely interventions for people with lower-limb functional dysfunction. Moreover, the application of wearable sensing, machine learning and artificial intelligence in this field enables accurate quantitative analysis of the relationship between walking speed and the biomechanics of the musculoskeletal system.^[72–75]^ Establishing accurate functional equations and mathematical models that link walking speed with the musculoskeletal system is essential for providing a scientific foundation for new technological applications. Therefore, an in-depth exploration of the working principles of the muscles and the influence of neural control and perception on walking speed is necessary.

Moreover, the changes in human motor abilities are complex and evolve over time. Therefore, a longitudinal study with controlled variables in which walking speed is assessed multiple times in the same participant under different motor capacity states can be instrumental in validating and optimizing walking speed as a reliable assessment indicator. This will facilitate the development of a walking speed assessment system appropriate for people of different ages and sexes with varying degrees of motor dysfunction. Furthermore, the rehabilitation training methods for individuals with motor dysfunction have gradually changed from manually guided posture training to robot-assisted training and more abundant sports exercises. In a follow-up study, longitudinal studies could focus on the participants’ specific skeletal and muscular conditions to evaluate the effectiveness of these training methods. Such investigations could explore inflection points in training outcomes, potential compensatory mechanisms, and long-term effects on the musculoskeletal and nervous systems. Our findings would provide critical data for the development of targeted clinical interventions.

### 4.4. Limitations

This study visualized and analyzed the literature on walking speed as an evaluation metric using CiteSpace to explore research progress and trends. However, this study has some limitations. First, we only analyzed literature from the Web of Science core database, and relevant studies were not included. Moreover, future studies should use meta-analyses to assess the reliability of data in the literature.

## 5. Conclusion

This review offers a comprehensive analysis of the research trends and hotspots in walking speed within the field of Sport Sciences using bibliometric methods. Walking speed is a key clinical indicator of lower-limb locomotor function. First, future research should primarily explore the relationship between walking speed and cognitive ability, neural control, and lower-limb muscles using mathematical or biomechanical modeling techniques. Second, more longitudinal studies are needed to better understand the mechanisms underlying the long-term effects of motor dysfunction and rehabilitation training on walking speed and the critical time points for the effectiveness of rehabilitation training on walking speed. Additionally, it is essential to design targeted experiments based on the characteristics of participants to provide precise experimental data that will assist clinicians in developing more tailored and effective clinical rehabilitation programs. In conclusion, this review systematically sorts the literature on walking speed as a research topic and analyzes its knowledge framework and global development trends. This study offers valuable insights to inspire researchers in this field.

## Acknowledgments

This study was supported by the National Natural Science Foundation of China (11502154). We are deeply grateful for the accessibility and functionality of the CiteSpace and Origin software, which greatly facilitated completing our bibliometric analysis.

## Author contributions

**Conceptualization:** Luming Yang, Yuan Liu.

**Formal analysis:** Yuan Liu, Xinye Liu.

**Funding acquisition:** Luming Yang.

**Methodology:** Luming Yang, Yuan Liu.

**Project administration:** Shiyang Yan.

**Software:** Yuan Liu, Xinye Liu.

**Supervision:** Mei Wu, Longbin Zhang, Shiyang Yan.

**Writing – original draft:** Luming Yang, Yuan Liu.

**Writing – review & editing:** Mei Wu, Longbin Zhang, Shiyang Yan.

## Supplementary Material


