# Supplementary material for: Lower-limb locomotor function studies using walking speed as an assessment indicator: A bibliometric review from 2014 to 2024
Source: Medicine (Baltimore). 2025 Jun 13;104(24):e42756. doi: 10.1097/MD.0000000000042756 (PMC12173329; doi:10.1097/MD.0000000000042756)
Supplement: Supplementary file 1 [file medi-104-e42756-s001.docx]

**Lower-limb Locomotor Function Studies Using Walking Speed as an Assessment Indicator: A Bibliometric Review from 2014 to 2024**

**Supplemental Tables**

Supplemental Table 1. The information of the country with more than 100 research articles in walking speed studies.

| Ranking | Country | Counts |
| --- | --- | --- |
| 1 | USA | 652 |
| 2 | AUSTRALIA | 135 |
| 3 | CANADA | 128 |
| 4 | ENGLAND | 127 |
| 5 | NETHERLANDS | 110 |
| 6 | JAPAN | 109 |
