# Supplementary material for: Lower-limb locomotor function studies using walking speed as an assessment indicator: A bibliometric review from 2014 to 2024
Source: Medicine (Baltimore). 2025 Jun 13;104(24):e42756. doi: 10.1097/MD.0000000000042756 (PMC12173329; doi:10.1097/MD.0000000000042756)
Supplement: Supplementary file 2 [file medi-104-e42756-s002.docx]

**Lower-limb Locomotor Function Studies Using Walking Speed as an Assessment Indicator: A Bibliometric Review from 2014 to 2024**

**Supplemental Tables**

Supplemental Table 2. The information of the institutions with more than 20 research articles in walking speed studies.

| Ranking | Institution | Countries | Counts |
| --- | --- | --- | --- |
| 1 | University of Delaware | USA | 37 |
| 2 | Vrije Universiteit Amsterdam | Netherlands | 34 |
| 3 | University of Florida | USA | 25 |
| 4 | Katholieke Universiteit Leuven | Belgium | 24 |
| 5 | University of Groningen | Netherlands | 23 |
| 6 | University of North Carolina System | USA | 23 |
| 7 | University of Pittsburgh | USA | 22 |
| 8 | Harvard Medical School | USA | 21 |
| 9 | University of Massachusetts | USA | 21 |
| 10 | University of Melbourne | Australia | 21 |
