# Supplementary figures and images for: Lower-limb locomotor function studies using walking speed as an assessment indicator: A bibliometric review from 2014 to 2024
Source: Medicine (Baltimore). 2025 Jun 13;104(24):e42756. doi: 10.1097/MD.0000000000042756 (PMC12173329; doi:10.1097/MD.0000000000042756)

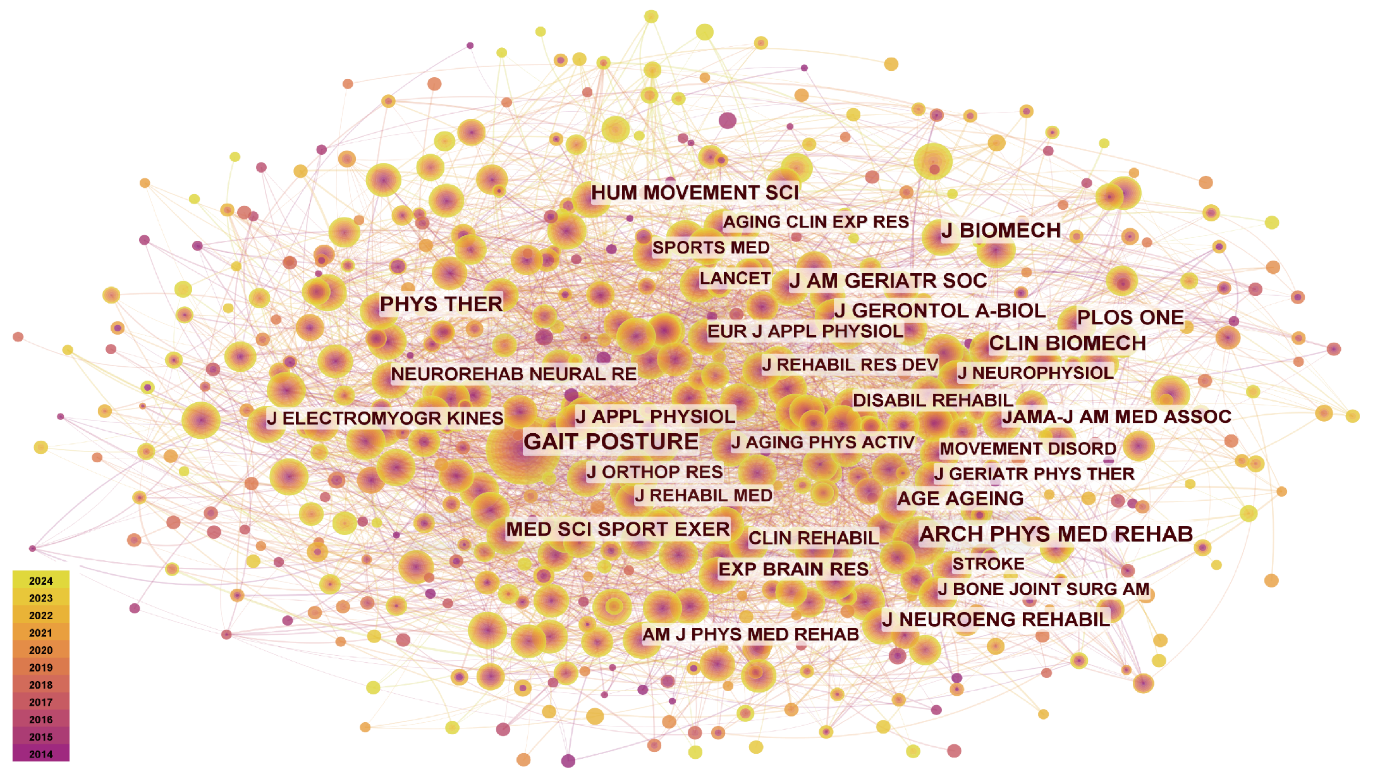

Supplement: Supplementary file 3 [file medi-104-e42756-s003.docx]
