# Supplementary material for: Lower-limb locomotor function studies using walking speed as an assessment indicator: A bibliometric review from 2014 to 2024
Source: Medicine (Baltimore). 2025 Jun 13;104(24):e42756. doi: 10.1097/MD.0000000000042756 (PMC12173329; doi:10.1097/MD.0000000000042756)
Supplement: Supplementary file 4 [file medi-104-e42756-s004.docx]

**Lower-limb Locomotor Function Studies Using Walking Speed as an Assessment Indicator: A Bibliometric Review from 2014 to 2024**

**Supplemental Tables**

Supplemental Table 3. The information of the authors with eight or more published articles in walking speed studies.

| Ranking | Name | Counts | Citations | Institution |
| --- | --- | --- | --- | --- |
| 1 | Franz, Jason R | 11 | 115 | University of North Carolina |
| 2 | Brach, Jennifer S | 9 | 155 | University of Pittsburgh |
| 3 | Bean, Jonathan F | 9 | 109 | Harvard Medical School |
| 4 | Perera, Subashan | 8 | 159 | University of Pittsburgh |
| 5 | Howell, David R | 8 | 182 | The Micheli Center for Sports Injury Prevention |
| 6 | Hortobagyi, Tibor | 8 | 144 | University of Groningen |
| 7 | Pietrosimone, Brian | 8 | 95 | University of North Carolina |
